# A metabolic marker–based diagnostic model for precancerous and malignant endometrial lesions in insulin-resistant PCOS women with sonographically suspected endometrial polyps

**DOI:** 10.3389/fonc.2026.1868252

**Published:** 2026-07-13

**Authors:** Ying Yang, Ningning Hu, Weimin Fan, Liwen Zhang, He Fei, Jun Ye, Yang Gao, Ju Yang, Jiangnan Pei, Rujun Chen

**Affiliations:** 1 Department of Obstetrics and Gynecology, Shanghai Fifth People’s Hospital, Fudan University, Shanghai, China; 2 Center of Community-Based Health Research, Fudan University, Shanghai, China; 3 Department of Pathology, Shanghai Fifth People’s Hospital, Fudan University, Shanghai, China; 4 Department of Obstetrics, Obstetrics and Gynecology Hospital of Fudan University, Shanghai, China

**Keywords:** diagnostic model, endometrial neoplasia, endometrial polyps, insulin resistance, polycystic ovary syndrome

## Abstract

**Background:**

Women with insulin-resistant polycystic ovary syndrome (PCOS-IR) and sonographically suspected endometrial polyps carry an elevated risk of endometrial premalignant and malignant lesions. This study aimed to characterize clinical and metabolic profiles across pathological subgroups and develop an exploratory risk-stratification model for endometrial neoplasia in this high-risk population, with a specific focus on insulin resistance as a core pathophysiological driver.

**Methods:**

A total of 185 PCOS-IR patients with ultrasound-detected endometrial polyps were retrospectively enrolled and stratified into benign (n=125), atypical hyperplasia (AH, n=34), and endometrial carcinoma (EC, n=26) groups. For modeling, AH and EC were combined into an endometrial neoplasia endpoint. A two-stage strategy combining LASSO regression and stepwise logistic regression was used for variable selection and model construction. Model performance was assessed via ROC curve, calibration curve, decision curve analysis, and multicollinearity diagnostics using the Variance Inflation Factor (VIF).

**Results:**

Compared with the benign group, the endometrial neoplasia group exhibited significantly higher HOMA-IR, fasting plasma glucose, 2-hour OGTT glucose, and fasting insulin, and significantly lower HDL-C (all *P*< 0.05). The final model incorporated age, high-density lipoprotein cholesterol (HDL-C), free androgen index (FAI), and homeostasis model assessment of insulin resistance (HOMA-IR), achieving moderate discrimination (AUC = 0.767) with high sensitivity (0.913) and specificity of 0.500. All variables had VIF values<5, confirming no significant multicollinearity. Good calibration and net clinical benefit were demonstrated in internal validation.

**Conclusion:**

Insulin resistance is linked to early metabolic alterations in PCOS-IR patients with endometrial polyps, and is a core component of the final predictive model. This four-variable model shows moderate discriminatory performance as an exploratory adjunctive tool for pre-hysteroscopy risk stratification, with high sensitivity to minimize missed diagnoses of endometrial neoplasia. Given the lack of external validation and the heterogeneity of the combined neoplasia endpoint, the model remains hypothesis-generating. Further external validation in independent cohorts is required before clinical application.

## 1 Introduction

Polycystic ovary syndrome (PCOS) is one of the most common endocrine–metabolic disorders affecting women of reproductive age, with a global prevalence of 5%–15% (1, 2). Insulin resistance (IR) is a core pathological feature of PCOS, present in 50%–70% of patients. Characterized by reduced glucose uptake and utilization under physiological insulin levels, IR leads to compensatory hyperinsulinemia, which further interacts with hyperandrogenemia to form a vicious cycle. This disruption promotes a spectrum of metabolic disorders including obesity, impaired glucose tolerance, dyslipidemia, chronic low−grade inflammation, metabolic syndrome, and type 2 diabetes mellitus (T2DM) (3–6).

Chronic anovulation in PCOS exposes the endometrium to prolonged unopposed estrogen stimulation without progesterone antagonism. Hyperinsulinemia and hyperandrogenemia secondary to IR further amplify this imbalance. The synergistic effects of unopposed estrogen, hyperinsulinemia, and hyperandrogenemia drive persistent abnormal endometrial proliferation, hyperplasia, and eventual malignant transformation (7–10). Compared with non−insulin−resistant PCOS, patients with insulin−resistant PCOS (PCOS−IR) display more severe metabolic disturbances and a significantly higher risk of endometrial cancer (11). The association between PCOS and endometrial cancer was first reported in 1957 (12), and IR−related metabolic–hormonal dysregulation is now recognized as the key mechanistic link (13–15).

Endometrial polyps (EPs) are localized overgrowths of endometrial tissue, with a prevalence of 10%–40% in women (14, 16). Although most EPs are benign, 3%–5% demonstrate premalignant or malignant changes (17, 18). Patients with PCOS, especially those with PCOS−IR, have a markedly higher incidence of EPs and an elevated risk of premalignant or malignant transformation (19). Thus, EPs may represent a critical intermediate lesion connecting PCOS−IR to endometrial carcinogenesis. However, the metabolic characteristics of EPs with different pathological outcomes in the PCOS−IR population remain poorly defined.

Current risk models for endometrial lesions are mainly derived from the general population and do not capture the unique metabolic and endocrine features of PCOS−IR, limiting their clinical utility in this high−risk group. Conventional diagnostic methods also have critical limitations: transvaginal ultrasound is prone to missed diagnoses, while hysteroscopy, the gold standard for EP detection, is invasive and cannot reliably distinguish benign from malignant lesions without histopathology. At present, no dedicated prediction model exists for identifying premalignant and malignant EPs specifically in PCOS−IR patients.

Accordingly, this retrospective cohort study was performed to compare clinical and metabolic profiles among PCOS−IR patients with benign, premalignant, and malignant endometrial polyps, identify key predictive markers, and establish an exploratory diagnostic model and provide a basis for future external validation. The goal is to provide a noninvasive, individualized risk stratification tool for early detection of high−risk EPs, thereby providing an exploratory risk−stratification tool and laying a foundation for further external validation.

## 2 Materials and methods

### 2.1 Study design and study population

This retrospective cohort study was conducted in accordance with the Declaration of Helsinki and approved by the Ethics Committee of Shanghai Fifth People’s Hospital (Approval No.: 2024−Lunshen−194). Owing to the retrospective nature of the study, the requirement for written informed consent was formally waived by the institutional review board. A total of 185 consecutive patients diagnosed with polycystic ovary syndrome (PCOS) accompanied by insulin resistance (IR) and histologically confirmed endometrial polyps were enrolled from the Department of Obstetrics and Gynecology at Shanghai Fifth People’s Hospital between March 2024 and December 2025. All patients underwent hysteroscopic polypectomy, and the final pathological diagnosis was established by two independent senior pathologists based on the resected specimens. Inclusion criteria are as follows: (1) diagnosis of PCOS was established according to the 2003 Rotterdam criteria, including oligo−ovulation or anovulation, clinical or biochemical hyperandrogenism, and polycystic ovarian morphology on ultrasound, with other etiologies excluded; (2) insulin resistance was defined as a homeostasis model assessment of insulin resistance (HOMA−IR) ≥ 2.69; (3) endometrial polyps were initially suspected by transvaginal ultrasound and subsequently confirmed by hysteroscopy and histopathological examination; (4) age ranged from 18 to 45 years old; (5) complete clinical, anthropometric, laboratory, imaging, surgical and pathological data were available for analysis. Exclusion criteria are as follows: (1) current pregnancy, lactation, or planned pregnancy within 3 months; (2) concurrent endocrine disorders including thyroid dysfunction, hyperprolactinemia, congenital adrenal hyperplasia, Cushing’s syndrome or other systemic metabolic diseases; (3) postmenopausal status; (4) history of endometrial ablation, hysterectomy, uterine malformation, or other malignant uterine diseases; (5) use of sex hormones, insulin sensitizers, oral contraceptives, or lipid−lowering medications within 3 months prior to enrollment. This study was a single−center retrospective analysis without an independent external validation cohort. Model generalizability requires further multi−center external validation.

### 2.2 Study grouping

Patients were divided into three groups according to postoperative histopathological findings, including benign group defined as benign endometrial polyps or simple endometrial hyperplasia without atypia, atypical hyperplasia (AH) group referring to premalignant lesions including complex hyperplasia with atypia, and endometrial carcinoma (EC) group representing histologically confirmed invasive endometrial carcinoma. For predictive modeling, AH and EC were combined into a single endometrial neoplasia endpoint. This grouping is clinically justified because both AH and EC require immediate intervention and close surveillance in PCOS− IR patients, representing a shared high−risk phenotype. Statistically, merging AH and EC improved event numbers (n=60) and stabilized model estimation, which is critical given the limited sample size. Nevertheless, this combined endpoint introduces biological and clinical heterogeneity, as AH is a premalignant lesion and EC is invasive carcinoma.

### 2.3 Data collection

#### 2.3.1 Demographic and clinical characteristics

General demographic and clinical information including age, menstrual status, reproductive history, and comorbidities were collected from standardized electronic medical records.

#### 2.3.2 Anthropometric measurements

All anthropometric assessments were performed by trained medical staff following a unified standard protocol. Height, weight, waist circumference and hip circumference were measured, and body mass index (BMI) and waist−hip ratio (WHR) were calculated accordingly.

#### 2.3.3 Laboratory measurements

All participants fasted for 8–12 hours overnight, and venous blood samples were collected between 7:00 and 9:00 a.m. Serum and plasma were separated within 30 minutes and stored at −80 °C until batch analysis. Glucose metabolism parameters included fasting plasma glucose (FPG), 1−h and 2−h plasma glucose during a standard 75−g oral glucose tolerance test (OGTT), and fasting insulin (FINS), and insulin resistance was calculated as HOMA−IR = (FPG × FINS)/22.5. Lipid profiles included triglycerides (TG), total cholesterol (TC), high− density lipoprotein cholesterol (HDL−C), and low−density lipoprotein cholesterol (LDL−C). Reproductive hormones included luteinizing hormone (LH), follicle−stimulating hormone (FSH), estradiol (E_2_), total testosterone (T), prolactin (PRL), and sex hormone−binding globulin (SHBG), and free androgen index (FAI) was calculated as FAI = (Total T/SHBG) × 100%.

### 2.4 Principal component analysis

Principal component analysis (PCA) was performed to visualize the overall clustering and separation trends of clinical, metabolic and endocrine profiles among patients with benign endometrial lesions, atypical hyperplasia and endometrial carcinoma, so as to reveal the overall distribution differences of multi−dimensional indicators across pathological subgroups. PCA was performed to visualize global multivariate separation across pathological groups and to inform variable selection by identifying correlated clusters prior to modeling.

### 2.5 Correlation analysis

Pearson or Spearman correlation analysis was performed to evaluate the pairwise linear relationships among candidate predictive indicators, and a correlation heatmap was used for visual display to judge the strength of correlation and identify potential multicollinearity among variables, so as to ensure the rationality of subsequent variable screening and model construction.

### 2.6 Variable selection by LASSO regression

Initial candidate predictors were selected based on established pathophysiological relevance rather than relying solely on intergroup statistical significance. Clinically meaningful variables (age, BMI, FAI) were retained for further evaluation despite nonsignificant group differences, given their recognized roles in PCOS−IR and endometrial neoplasia. Conversely, several variables with significant univariate differences (e.g., PRL, FINS, OGTT−2h, LH) were excluded *a priori* due to strong multicollinearity with core insulin resistance markers, to avoid collinearity bias in regression modeling.

Least absolute shrinkage and selection operator (LASSO) penalized logistic regression with ten−fold cross−validation was applied for aggressive dimensionality reduction and removal of collinear redundant variables, using the full six-variable candidate pool (age, BMI, HOMA_IR, FPG, HDL_C and FAI). The optimal penalty parameter λ was determined by ten−fold cross−validation to balance model fitness and complexity, identifying HDL−C as the most robust independent predictor. Notably, LASSO inherently penalizes clinically meaningful but weakly predictive variables toward zero, which may exclude biologically relevant factors. Given the retrospective single-center design, the total number of neoplastic events was limited (n = 60), resulting in an events-per-variable ratio at the lower bound of recommended standards. This increases the risk of model instability and overfitting. Therefore, we adopted a two−stage parsimonious strategy combining LASSO and stepwise regression: LASSO filtered redundant variables, followed by bidirectional stepwise regression to re−evaluate and retain variables with established pathophysiological relevance and independent prognostic value, thereby balancing statistical rigor and clinical interpretability.

### 2.7 Predictive model construction by stepwise logistic regression

Based on the two−stage variable selection strategy, bidirectional stepwise logistic regression was performed following LASSO screening to construct the final diagnostic model distinguishing benign endometrial lesions from endometrial neoplasia. This step aimed to re−evaluate variables with known pathophysiological relevance (age and FAI) that were penalized by LASSO, retaining those with independent statistical significance and established clinical plausibility. The odds ratio (OR), 95% confidence interval (CI), and corresponding *P*−value were calculated for each independent predictor included in the final model.

### 2.8 Nomogram construction

A visual diagnostic nomogram was established based on the final four−variable logistic regression model, so as to transform the regression coefficients into intuitive scoring scales. Each variable was assigned a corresponding score, and the total score of each patient was mapped to the corresponding predicted probability of endometrial neoplasia, so as to realize the individualized visual risk calculation for clinical application.

### 2.9 Model discrimination by ROC curve analysis

The discrimination ability of the model was evaluated by receiver operating characteristic (ROC) curve analysis, and the area under the ROC curve (AUC) was calculated to quantify the overall differentiation efficiency. The optimal cutoff value was determined according to the Youden index, and the corresponding sensitivity and specificity were calculated to reflect the diagnostic accuracy of the model.

### 2.10 Model calibration by calibration curve

Calibration curves with 1000 bootstrap resamples were drawn to evaluate the consistency between the predicted probability of the model and the actual pathological outcome. The proximity between the calibration curve and the ideal diagonal line was used to judge the calibration degree of the model, so as to verify whether the predicted risk was consistent with the actual observed risk.

### 2.11 Clinical utility by decision curve analysis

Decision curve analysis (DCA) was performed to evaluate the net clinical benefit of the prediction model within the range of clinically reasonable threshold probabilities. By comparing the net benefit of the model with that of treating all patients or treating none, the clinical application value and clinical net benefit of the model were comprehensively evaluated.

### 2.12 Basic statistical processing

Basic statistical analyses were performed using SPSS 26.0 (IBM Corp., Armonk, NY, USA) and R software (version 4.2.1; http://www.r-project.org/) with the ggplot2, corrplot, glmnet, rms, pROC and rmda packages. Continuous variables were presented as mean ± standard deviation (SD) or median (interquartile range, IQR) according to data distribution, and between−group comparisons were conducted using one−way ANOVA or Kruskal–Wallis H test. Categorical variables were presented as frequencies or percentages and compared using the χ² test or Fisher’s exact test. All statistical tests were two−sided, and *P*< 0.05 was considered statistically significant. For *post-hoc* pairwise comparisons following the Kruskal-Wallis H test, the Dunn-Bonferroni correction was applied to account for multiple comparisons and control the overall type I error rate. This study adhered to the Transparent Reporting of a multivariable prediction model for Individual Prognosis Or Diagnosis (TRIPOD) guidelines. Missing data were handled via complete−case analysis, with no variable exceeding 5% missing values. Model calibration was assessed using 1000 bootstrap resamples to derive optimism−corrected calibration slopes and intercepts. The final model equation, regression coefficients, and random seed for reproducibility were documented to facilitate independent validation.

## 3 Results

### 3.1 Clinical and demographic characteristics

A total of 185 patients were enrolled in this study, including 125 with benign endometrial lesions, 34 with atypical hyperplasia (AH), and 26 with endometrial carcinoma (EC). Baseline clinical, metabolic, and hormonal profiles were compared across the three groups (**Table 1**). No significant differences in age (*P* = 0.791) or body mass index (BMI, *P* = 0.181) were observed, indicating comparable demographic and general obesity distributions. For glycometabolic and insulin resistance markers, significant intergroup differences were detected in homeostasis model assessment of insulin resistance (HOMA-IR, *P* = 0.031), fasting plasma glucose (FPG, *P* = 0.025), 2-hour oral glucose tolerance test (OGTT-2h, *P* = 0.049), and fasting insulin (FINS, *P* = 0.021). *Post-hoc* analysis showed that the AH group had significantly higher HOMA-IR, FPG, OGTT-2h, and FINS levels than the benign group (all *P*< 0.05). No significant differences were found between AH and EC or between benign and EC (all *P* > 0.05). For lipid profiles, high-density lipoprotein cholesterol (HDL-C) differed significantly among groups (*P* = 0.005). The AH group had significantly lower HDL-C than the benign group (*P* = 0.010), while no difference was observed between AH and EC (*P* = 0.060) or between benign and EC (*P* > 0.05). Triglycerides (TG), total cholesterol (TC), and low-density lipoprotein cholesterol (LDL-C) were comparable across all groups (all *P* > 0.05). For reproductive hormones, luteinizing hormone (LH, *P* = 0.039) and prolactin (PRL, *P* = 0.005) showed significant intergroup differences. LH was significantly lower in the AH group versus the benign group (*P* = 0.039). PRL was significantly higher in the EC group than in both the benign and AH groups (both *P* = 0.006). No significant differences were noted for follicle-stimulating hormone (FSH), estradiol (E2), 17α-hydroxyprogesterone (OH17P), anti-Müllerian hormone (AMH), or free androgen index (FAI) (all *P* > 0.05). Notably, although AH and EC were combined as an endometrial neoplasia endpoint for modeling, biological heterogeneity between premalignant and malignant lesions should be carefully interpreted. For the 26 patients with EC, all cases were endometrioid adenocarcinoma, grade 1 (G1), and FIGO stage I disease.

**Table 1:** Clinical and demographic characteristics of the Benign, AH, and EC groups (n=185).

Variable	Benign	AH	EC	P_overall	P_Benign vs AH	P_Benign vs EC	P_AH vs EC
n	125	34	26				
Age (yrs)	31.88 (5.27)	31.21 (4.72)	31.88 (4.11)	0.791			
BMI	26.7 (5.2)	28.8 (5.21)	28.08 (5.89)	0.181			
HOMA_IR	3.54 (2.39)	5.91 (4.80)	5.57 (6.11)	0.031	0.009	0.375	0.172
FPG (mmol/L)	5.03 (0.57)	5.6 (1.76)	5.46 (1.51)	0.025	0.041	0.885	0.188
HDL_C (mmol/L)	1.39 (0.43)	1.14 (0.22)	1.19 (0.25)	0.005	0.01	0.885	0.06
FAI	8.64 (6.63)	9.72 (9.40)	6.34 (6.58)	0.371			
OGTT_2h (mmol/L)	7.46 (2.4)	9.30 (4.24)	8.24 (3.53)	0.049	0.043	0.498	0.555
FINS (pmol/L)	92.33 (55.56)	136.81 (94.83)	122.62 (100.84)	0.021	0.027	0.781	0.208
FSH (mIU/mL)	6.64 (2.13)	5.62 (1.93)	6.52 (2.05)	0.08			
LH (mIU/mL)	10.58 (6.7)	7.22 (4.33)	8.7 (6.54)	0.039	0.039	0.694	0.433
E2 (pmol/L)	189.31 (180.17)	183.77 (130.88)	157.52 (86.54)	0.729			
PRL (mIU/mL)	274.28 (149.92)	232.13 (123.32)	598.11 (982.92)	0.005	0.841	0.006	0.006
OH17P (ng/mL)	1.02 (1.88)	0.89 (0.9)	0.95 (0.59)	0.977			
AMH (ng/mL)	8.3 (5.92)	7.15 (5.41)	9.41 (6.19)	0.398			
TG (mmol/L)	1.55 (0.76)	1.83 (1.12)	1.43 (0.89)	0.231			
TC (mmol/L)	4.88 (0.97)	4.95 (0.97)	4.88 (0.8)	0.953			
LDL_C (mmol/L)	3.28 (0.88)	3.54 (1.03)	3.43 (0.86)	0.46			

AH, atypical hyperplasia; AMH, anti-Müllerian hormone; BMI, body mass index; EC, endometrial carcinoma; E2, estradiol; FAI, free androgen index; FINS, fasting insulin; FPG, fasting plasma glucose; FSH, follicle-stimulating hormone; HDL-C, high-density lipoprotein cholesterol; HOMA-IR, homeostasis model assessment of insulin resistance; LH, luteinizing hormone; LDL-C, low-density lipoprotein cholesterol; OGTT-2h, 2-hour oral glucose tolerance test; OH17P, 17α-hydroxyprogesterone; PRL, prolactin; TC, total cholesterol; TG, triglycerides.

### 3.2 Multivariate distribution and correlation of clinical metabolic markers

**Figure 1:**
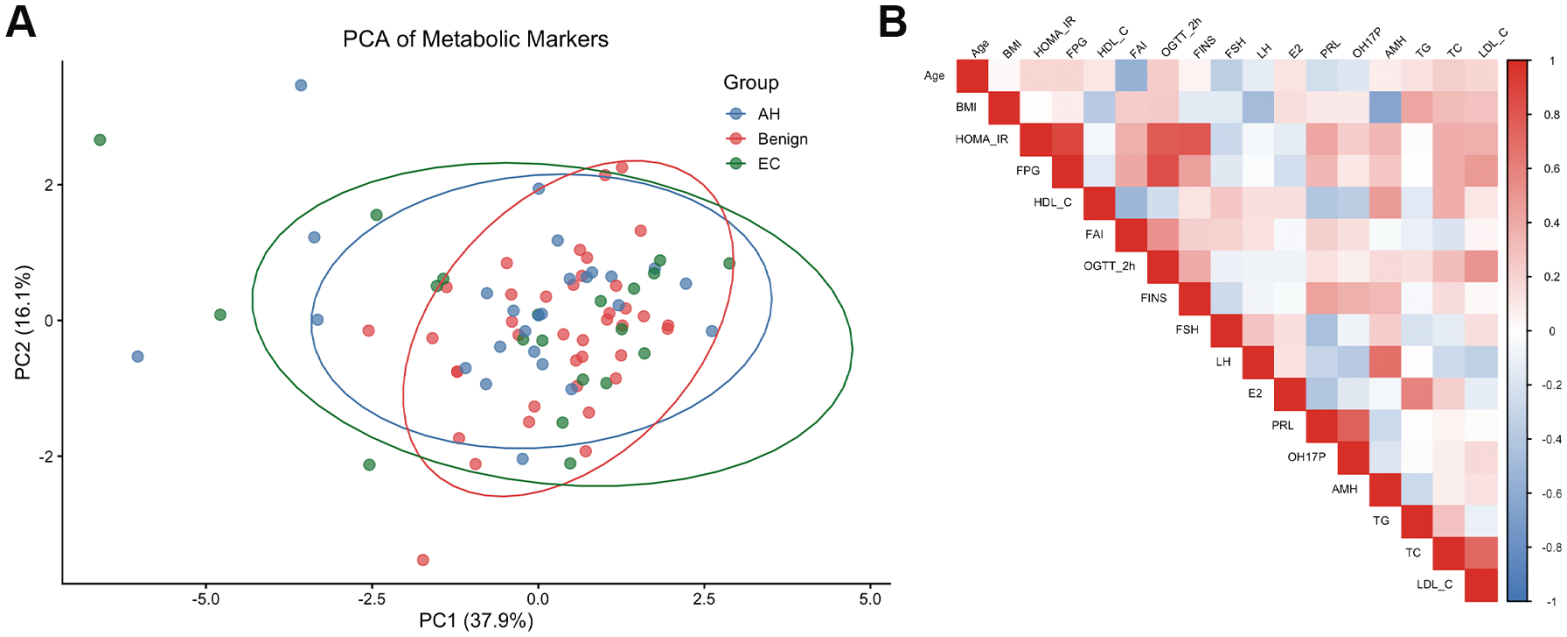
Multivariate profiling and correlation of clinical and metabolic markers. **(A)** Principal component analysis (PCA) of the included metabolic markers across three pathological groups. Blue dots: atypical hyperplasia (AH); red dots: benign endometrial lesions; green dots: endometrial carcinoma (EC). PC1 accounts for 37.9% and PC2 accounts for 16.1% of the total variation. Ellipses denote group distribution ranges. **(B)** Correlation heatmap showing pairwise relationships among demographic, metabolic and reproductive endocrine variables. Red shading represents positive correlations, blue shading represents negative correlations, with color depth corresponding to the strength of correlation coefficient.

To characterize the overall multivariate distribution of clinical and metabolic profiles across different pathological statuses, principal component analysis (PCA) was performed on the full panel of included markers. As shown in **Figure 1A**, patients with benign endometrial lesions exhibited a distinct clustering pattern relative to those with premalignant and malignant endometrial neoplasia. While partial overlap was observed between the atypical hyperplasia (AH) and endometrial carcinoma (EC) subgroups, a clear separation trend was identified when comparing benign versus combined neoplastic cases, indicating systematic alterations in metabolic signatures associated with disease progression.

Correlation heatmap analysis was subsequently conducted to evaluate pairwise associations among all demographic, glycometabolic, lipometabolic and hormonal parameters. As illustrated in **Figure 1B**, a series of moderate and biologically plausible correlations were detected between age, insulin resistance indices, lipid profiles and reproductive endocrine markers. Notably, all correlation coefficients were below 0.7, and no severe multicollinearity was observed across variables, supporting the reliability and rationality of subsequent predictive model construction.

### 3.3 Intergroup difference of core insulin resistance phenotype and establishment of diagnostic modeling

**Figure 2:**
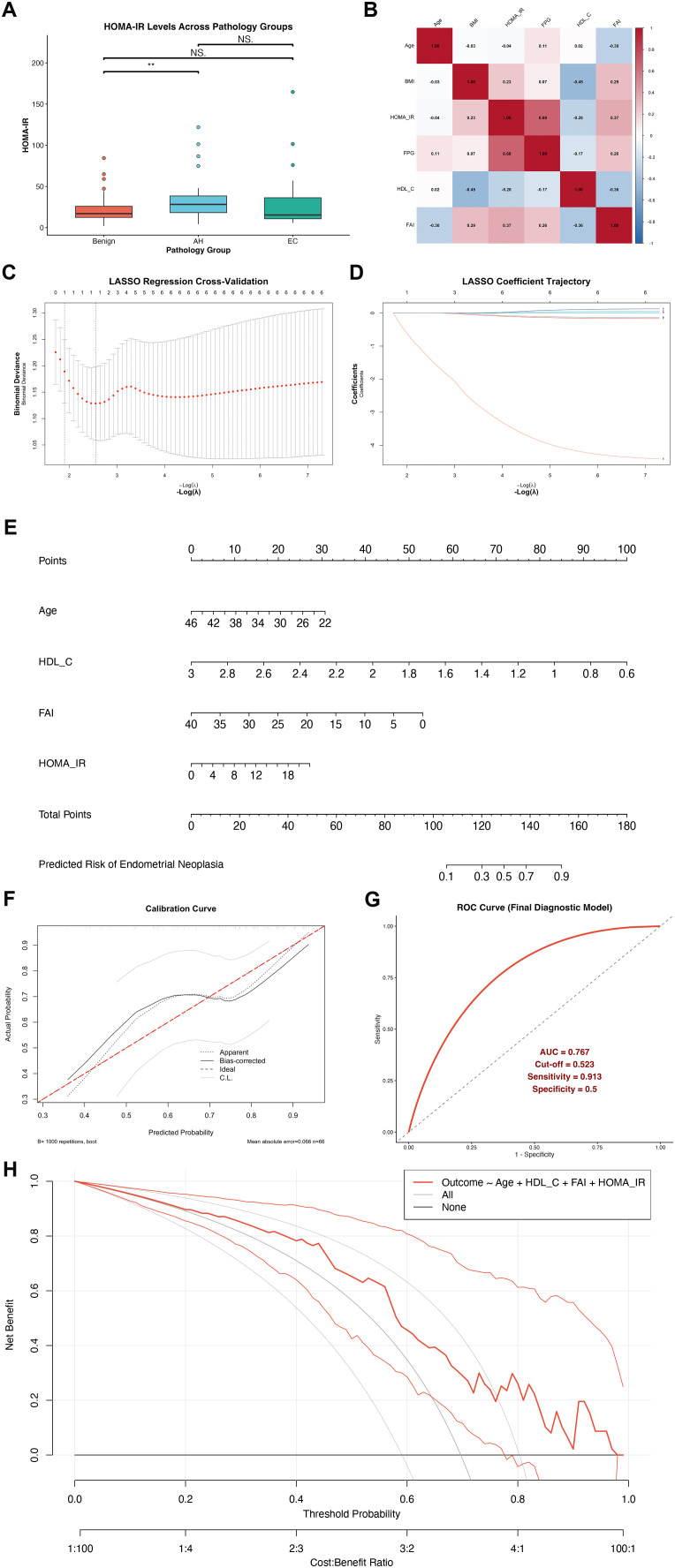
Phenotypic comparison, variable screening, nomogram construction and performance evaluation of the diagnostic prediction model. **(A)** Distribution of HOMA−IR levels among the benign endometrial lesion, atypical hyperplasia (AH), and endometrial carcinoma (EC) groups. **P < 0.01; NS, no significant difference. **(B)** Correlation heatmap of the six candidate predictive variables. Red shades indicate positive correlations, blue shades indicate negative correlations, and color intensity corresponds to the strength of the correlation coefficient. **(C)** Ten−fold cross−validation curve of the LASSO regression for determination of the optimal penalty parameter λ. **(D)** LASSO coefficient trajectory plot showing the variation of variable coefficients with changing λ values. **(E)** Nomogram of the final predictive model for individualized risk estimation of endometrial neoplasia. **(F)** Calibration curve of the diagnostic model. Apparent, unadjusted calibration curve; Bias−corrected, bootstrap internal calibration curve; Ideal, reference perfect prediction line; C.L., confidence limit. A total of 1000 bootstrap resamples were performed. **(G)** Receiver operating characteristic (ROC) curve of the final diagnostic model, with corresponding AUC, optimal cutoff value, sensitivity, and specificity displayed. **(H)** Decision curve analysis (DCA) comparing the net clinical benefit of the established nomogram with the “treat all” and “treat none” strategies across varying threshold probabilities.

Comparison of HOMA-IR levels across the three pathological subgroups demonstrated that insulin resistance was significantly elevated in patients with atypical hyperplasia compared with those diagnosed with benign endometrial lesions (*P*< 0.01), as presented in **Figure 2A**. No statistically significant difference was observed between the AH and EC groups. Considering the similar high-risk biological characteristics and clinical management implications of premalignant and malignant endometrial lesions, we grouped AH and EC cases into a single composite endpoint of endometrial neoplasia for subsequent diagnostic modelling.

The primary modelling objective was to develop a predictive tool capable of distinguishing benign endometrial pathology from combined endometrial neoplasia, specifically among insulin-resistant PCOS patients with ultrasound-confirmed endometrial polyps. Based on clinical pathophysiological relevance and univariate screening results, six baseline variables were initially selected as candidate predictors for model development: age, BMI, HOMA-IR, fasting plasma glucose (FPG), HDL-C and free androgen index (FAI). Pairwise correlation analysis of the six candidate predictors was performed and presented in the correlation heatmap (**Figure 2B**). There was a strong positive correlation between HOMA_IR and FPG (*r* = 0.68), while moderate negative correlations were observed between BMI and HDL_C (*r* = -0.45), HDL_C and FAI (*r* = -0.36), as well as between Age and FAI (*r* = -0.30). Several moderate positive correlations were also noted among metabolic and hormonal indicators. Notably, all correlation coefficients were below 0.7, and no significant multicollinearity was detected between variables, which supported the reliability and rationality of subsequent predictive model construction.

### 3.4 Variable selection and final predictor identification

To minimize model overfitting and achieve rigorous dimensional reduction, LASSO penalized binary logistic regression was first applied for automated feature selection. Ten-fold cross-validation was used to determine the optimal penalty λ value, which balanced model deviance and complexity. As depicted in **Figure 2C** (LASSO Regression Cross-Validation) and **Figure 2D** (LASSO Coefficient Trajectory), HDL-C remained the only stable non-zero coefficient at the optimal lambda, while other candidate variables were effectively penalized and shrunk towards zero, indicating limited independent predictive contribution beyond HDL-C.

Following LASSO screening, bidirectional stepwise multivariate logistic regression was further applied, incorporating clinically meaningful variables including HOMA-IR, which is central to the insulin−resistance focus of this study. The final predictive model retained age, HDL-C, FAI, and HOMA-IR, all of which were clinically and pathophysiologically relevant. Multicollinearity diagnostics using variance inflation factor (VIF) confirmed that all variables had VIF< 5, indicating no significant multicollinearity (**Supplementary Table 1**).

The final multivariable logistic regression equation was:


Logit(P) = 9.9601 − 0.1183 × Age − 3.8497 × HDL−C − 0.1227 × FAI + 0.1142 × HOMA−IR

The predicted probability of endometrial neoplasia was calculated as:


P = 1/(1 + exp(−(9.9601 − 0.1183 × Age − 3.8497 × HDL−C − 0.1227 × FAI + 0.1142 × HOMA−IR)))

Among the final predictors, reduced HDL−C was a significant independent protective factor, while elevated HOMA−IR showed a trend toward increased risk. Age and FAI exhibited marginal modifying effects. Owing to the relatively small number of neoplastic events (n = 60), the final model was intentionally restricted to a parsimonious set of predictors to minimize overfitting risk. Full regression results for the final four-variable model are provided in **Supplementary Table 2**.

### 3.5 Construction and validation of the diagnostic nomogram

Based on the final four−variable regression framework, an individualized visual nomogram was established to facilitate convenient clinical risk estimation. As shown in **Figure 2E**, each predictor was assigned a corresponding point score, and the summed total points could be readily converted into a personalized predicted probability of developing endometrial neoplasia.

Calibration curve analysis demonstrated favorable agreement between model−predicted probabilities and actual observed outcomes, with minimal deviation after internal bootstrap correction. As presented in **Figure 2F**, the calibration curve indicated good consistency between predicted and actual probabilities. Receiver operating characteristic (ROC) curve analysis further revealed that the final model achieved moderate discrimination (AUC = 0.767) in the target high−risk population. At the optimal Youden−derived cut− off value of 0.523, the model yielded high sensitivity (0.913) and specificity of 0.500 for the detection of endometrial neoplasia (**Figure 2G**), reflecting a clinical trade−off to minimize false negatives. Decision curve analysis (**Figure 2H**) confirmed that the model provided a net clinical benefit over the strategies of treating all or treating none across a reasonable threshold range. Collectively, these findings indicate that this age−HDL−C−FAI−HOMA−IR−based nomogram has acceptable discrimination and calibration, serving as an exploratory adjunctive tool for preoperative risk stratification in this specific patient cohort.

## 4 Discussion

In the present study, HOMA-IR was significantly higher in the atypical hyperplasia (AH) group than in the benign endometrial polyp group, whereas no significant difference was observed between the AH and endometrial carcinoma (EC) groups. These findings are consistent with previous evidence indicating that insulin resistance constitutes a core driver of endometrial hyperplasia and malignant transformation in women with PCOS (19–24). The plateauing of systemic insulin resistance from AH to EC is consistent with previous reports indicating that malignant endometrial cells may exhibit reduced dependence on systemic insulin stimulation (25–30). Correlation analysis further demonstrated that HOMA-IR was positively correlated with FPG and FAI, and inversely correlated with HDL-C, supporting the notion that insulin resistance interacts with glycolipid metabolic disturbance and hyperandrogenism to create a pro-proliferative and pro-inflammatory intrauterine microenvironment that promotes the malignant transformation of endometrial polyps (21, 31).

The nomogram established in this study exhibited a unique predictor weighting structure that reflects the specific pathophysiology of PCOS-IR rather than conventional risk patterns derived from the general population. Younger age was associated with an increased predicted risk score, which differs from the linear age-dependent risk observed in the general population but is biologically plausible in PCOS-IR patients, who are exposed to prolonged chronic anovulation, unopposed estrogen stimulation, and insulin resistance from early reproductive age (7–10). Lower HDL-C corresponded to a higher risk score, in line with published evidence indicating that HDL-C exerts anti-inflammatory, antioxidant, and anti-proliferative effects, and that reduced HDL-C is associated with elevated gynecological malignancy risk (32–34). The inverse association between FAI and neoplastic risk observed in this cohort may reflect the dominant influence of severe metabolic dysfunction over isolated hyperandrogenism in PCOS-IR-related endometrial carcinogenesis, which differs from the positive association reported in general-population studies (35, 36). Meanwhile, BMI, FPG, and HOMA-IR showed relatively modest point contributions in the nomogram, likely due to multicollinearity among these interrelated metabolic markers, rather than a lack of biological relevance. We acknowledge that the directions of association for age and FAI are counterintuitive and lack univariate support in **Table 1**. Younger age and lower FAI were associated with higher neoplastic risk only in the multivariable model, likely reflecting multivariate adjustment effects and confounding by metabolic factors. Given the limited sample size and single-center design, these findings may be subject to overfitting or chance associations; the biological interpretations are hypothesis-generating and require external validation in larger cohorts.

Methodologically, we adopted a two−stage variable selection strategy to balance statistical rigor and clinical interpretability. LASSO regression was used for initial aggressive dimensionality reduction, identifying HDL−C as the dominant predictor; however, LASSO inherently penalizes weakly predictive but pathophysiologically meaningful variables. Subsequent bidirectional stepwise regression re−evaluated and retained age and FAI, which are closely linked to prolonged unopposed estrogen exposure and hyperandrogenism in PCOS−IR. This approach avoids arbitrary data−driven selection bias and enhances the clinical interpretability of the final model.

We acknowledge that combining atypical hyperplasia (AH) and endometrial carcinoma (EC) into a single endpoint introduces biological and clinical heterogeneity. AH represents a premalignant state with potential regression, while EC is an invasive malignancy with distinct prognosis and treatment. However, this grouping is clinically meaningful for PCOS−IR patients, in whom both conditions warrant urgent hysteroscopy and intensified metabolic management. From a statistical perspective, merging AH and EC increased the number of positive events (n=60), reducing model instability caused by the small EC subgroup (n=26). Despite these advantages, the heterogeneity of the combined endpoint may attenuate model specificity and limit direct extrapolation to pure EC or pure AH populations. Future studies with larger cohorts should evaluate the model separately for AH and EC to clarify its performance in distinct pathological stages.

Regarding model performance and clinical utility, the present nomogram showed moderate discriminative ability (AUC = 0.767), high sensitivity (0.913), and specificity of 0.500. High sensitivity ensures minimal false negatives, a critical goal for identifying neoplastic lesions in women already at elevated baseline risk. The model achieves high sensitivity of 0.913 accompanied by a specificity of 0.500, which inevitably generates a certain number of false-positive predictions. False-positive findings may trigger patient anxiety, unnecessary psychological stress and extra clinical examinations, which bring tangible adverse impacts to patients. Although these patients will receive standardized routine gynecologic follow-up after risk stratification, regular surveillance can only partially relieve the clinical adverse consequences caused by false positives rather than completely eliminate them. Clinically, the model functions as a non-invasive pre-hysteroscopy risk-stratification tool: it prioritizes urgent hysteroscopy for high-score patients and supports watchful waiting for low-score patients, thereby optimizing resource allocation and reducing unnecessary invasive procedures. Importantly, the model is not intended to replace hysteroscopy or histopathology; it is an exploratory adjunctive tool to aid clinical decision-making in this specific high-risk population.

Compared with existing endometrial lesion risk prediction models, the current model has several distinctive strengths. Most published models are based on the general or postmenopausal population and rely heavily on age, menopausal status, and endometrial thickness on ultrasound (37, 38), and thus cannot adequately reflect the specific metabolic-endocrine phenotype of PCOS-IR patients. Other models include generic metabolic risk factors without emphasizing insulin resistance as the central pathogenic mediator. By contrast, the present model was specifically developed for PCOS-IR patients with endometrial polyps, uses only routine laboratory parameters, and captures the unique risk distribution of this population. The model exhibited acceptable discrimination, calibration, and net clinical benefit in the internal validation; however, these results require confirmation in external cohorts. At present, the model remains exploratory and hypothesis−generating. Emerging research also highlights novel biomarkers such as kallistatin, which reflects metabolic and inflammatory dysregulation in PCOS and may provide additional insights into PCOS-related endometrial dysfunction (39).

Several limitations of this study should be noted. First, this was a single−center retrospective study with a limited number of neoplastic events (n = 60), reaching the lower bound of the recommended events-per-variable ratio for multivariable prediction modeling. The sample sizes of the atypical hyperplasia (AH, n=34) and endometrial carcinoma (EC, n=26) subgroups were particularly small, resulting in insufficient statistical power for direct comparative analysis between these two pathological types, thus we only performed preliminary exploratory intergroup comparisons rather than rigorous differential modeling. To mitigate associated statistical bias and overfitting risk, we intentionally restricted the final model to four core predictors, but inherent limitations of small subgroup sizes still exist. Second, no independent external validation or temporal validation cohort was included, and model evaluation relied solely on internal bootstrap validation, which may lead to relatively optimistic performance estimates and restrict population generalizability, so this model can only be regarded as exploratory research result and cannot be directly applied in clinical practice temporarily. Third, detailed pathological information including FIGO stage, histological grade and histological subtype of endometrial carcinoma was not incorporated into the main statistical analysis, and the benign group in this study combined two different clinical entities including benign endometrial polyps and simple endometrial hyperplasia without atypia; relevant sensitivity analysis by separating these two subgroups could not be conducted due to limited sample size after further grouping. Fourth, the model was designed to distinguish benign lesions from combined endometrial neoplasia (AH+EC), and its ability to differentiate AH from EC alone is insufficient, and future studies can improve discriminative accuracy by incorporating imaging omics, molecular biomarkers or long-term follow-up data. Fifth, the cohort was restricted to PCOS−IR patients with ultrasound−suspected endometrial polyps who underwent hysteroscopy and histopathology, introducing certain selection bias and spectrum bias, and this enriched high−risk population may artificially inflate observed model performance and limit generalizability to unselected PCOS−IR patients without sonographic polyp suspicion.

## 5 Conclusion

In conclusion, insulin resistance is closely associated with the progression of endometrial polyps toward premalignant and malignant lesions in insulin−resistant PCOS patients. The present metabolic nomogram including age, HDL−C, FAI, and HOMA−IR showed promising discriminatory and calibrated performance in the study cohort. However, due to the lack of external validation, the model should be considered exploratory and hypothesis−generating rather than clinically applicable. Further external validation in independent cohorts is required before its clinical use.

## Data Availability

The original contributions presented in the study are included in the article/**Supplementary Material**. Further inquiries can be directed to the corresponding authors.
