# Supplementary material for: A metabolic marker–based diagnostic model for precancerous and malignant endometrial lesions in insulin-resistant PCOS women with sonographically suspected endometrial polyps
Source: Front Oncol. 2026 Jul 13;16:1868252. doi: 10.3389/fonc.2026.1868252 (PMC13402187; doi:10.3389/fonc.2026.1868252)
Supplement: Supplementary file 1 [file Supplementaryfile1.docx]

Supplementary Table 1. VIF collinearity diagnosis results for the endometrial neoplasia prediction model

| Variable | VIF_Value | Collinearity_Level |
| --- | --- | --- |
| Age | 1.337 | No Collinearity |
| HDL_C | 1.419 | No Collinearity |
| FAI | 1.935 | No Collinearity |
| HOMA_IR | 1.229 | No Collinearity |
