# Supplementary material for: A metabolic marker–based diagnostic model for precancerous and malignant endometrial lesions in insulin-resistant PCOS women with sonographically suspected endometrial polyps
Source: Front Oncol. 2026 Jul 13;16:1868252. doi: 10.3389/fonc.2026.1868252 (PMC13402187; doi:10.3389/fonc.2026.1868252)
Supplement: Supplementary file 2 [file Supplementaryfile2.docx]

Supplementary Table 2. Multivariable logistic regression results for the four-variable endometrial neoplasia risk prediction model in PCOS-IR patients

| Variable | OR | CI_95_Lower | CI_95_Upper | P_Value |
| --- | --- | --- | --- | --- |
| Age | 0.888 | 0.754 | 1.029 | 0.129 |
| HDL-C | 0.021 | 0.001 | 0.27 | 0.0083 |
| FAI | 0.885 | 0.782 | 0.983 | 0.0318 |
| HOMA-IR | 1.121 | 0.946 | 1.402 | 0.2362 |
